# Impact of glucose dosing regimens on modeling of glucose tolerance and *β*‐cell function by intravenous glucose tolerance test in diet‐induced obese mice

**DOI:** 10.14814/phy2.12011

**Published:** 2014-05-20

**Authors:** Bilal A. Omar, Giovanni Pacini, Bo Ahrén

**Affiliations:** 1 Department of Clinical Sciences, Lund University, Lund, Sweden; 2 Metabolic Unit, Institute of Biomedical Engineering (ISIB‐CNR), Padova, Italy

**Keywords:** Diet‐induced obesity, insulin secretion, insulin sensitivity, mathematical modeling

## Abstract

Insulin sensitivity declines in overweight and obese individuals and, under normal conditions, insulin secretion adaptively increases which in healthy non‐diabetic subjects maintains normal glycemia. This adaptation is best described by the disposition index derived from modeling of insulin and glucose data from an intravenous glucose tolerance testing (IVGTT). One caveat of the IVGTT is that basing the glucose dose on the individual total body weight can result in large differences in the amount of glucose given to lean and obese individuals. The effect this has on determination of insulin sensitivity and *β*‐cell function is unknown. In this study, we therefore evaluated alternative glucose dosing regimens for determination of the impact of glucose dosing on measures of *β*‐cell function in normal and diet‐induced obese (DIO) mice. The glucose dosing regimens used for the IVGTT were 0.35 mg per kg total body weight (BW) or per kg lean BW or a fixed glucose dose based on the average BW for all experimental mice. Each regimen detected a similar decrease in insulin sensitivity in DIO mice. The different glucose dosing regimens gave, however, diverging results in regard to glucose elimination and the acute insulin response. Thus, the fixed‐dose regimen was the only that revealed impairment of glucose elimination, whereas dosing according to total BW was the only regimen which showed significant increases in acute insulin response in DIO mice. The fixed‐dose glucose dosing regimen was the only that revealed a significant decline in the disposition index value in DIO mice, which is characteristic of type 2 diabetes in humans. Our results therefore show that using different glucose dosing regimens during IVGTT in DIO mice one can model different aspects of physiology which are similar to prediabetes and type 2 diabetes in humans, with the fixed‐dose regimen producing a phenotype that most closely resembles human type 2 diabetes.

## Abstract (precis)

It is unclear what effect the amount of glucose given during an intravenous glucose tolerance test has on measures of glucose tolerance in diet‐induced obese mice. We tested glucose dosing based on total body weight, lean body mass, or a body weight‐independent fixed dose. The fixed‐dose glucose dosing regimen was the only that revealed impaired insulin sensitivity, insufficient insulin secretion, and a diminished disposition index, all hallmarks of type 2 diabetes, and is thus the optimal glucose dosing regimen for intravenous glucose tolerance testing in diet‐induced obese mice.

## Introduction

Intravenous glucose tolerance tests (IVGTT) quantify insulin secretion and estimate insulin sensitivity with the aid of mathematical modeling (Galvin et al. 1992). These parameters can therefore be used to quantify the mechanism for which increased *β*‐cell function compensates for an augmented insulin resistance. This quantification is made by the disposition index, which is based on the hyperbolic relationship between insulin sensitivity and insulin secretion and, mathematically, is described as the product of insulin sensitivity multiplied by insulin secretion (Bergman et al. 1981; Kahn et al. 1993). The use of IVGTT has therefore been of value to evaluate *β*‐cell function in various degrees of insulin resistance, such as comparing normal weight and obese individuals (Lorenzo et al. 2010). Since glucose is administered in a dose depending on body weight, an oft arising question is whether different glucose loads could yield different results in the same individual. This is also very important when results between normal weight and obese individuals are to be compared. For example, dosing the glucose load by body weight may result in large variation in glucose load between individuals. Conversely, dosing of the glucose load equally in all individuals, regardless of body weight, may result in large differences in peak glucose levels thereby affecting both insulin secretion and the assessment of insulin sensitivity. The dosing strategy may therefore per se contribute to the results and the conclusions of studies on islet function in relation to insulin sensitivity. More than 30 years ago, it was determined that giving different oral doses of glucose to individuals with impaired glucose tolerance resulted in different evaluation of their glycemic status (de Nobel and van't Laar 1978). This resulted in standardization of the oral glucose load to 75 g as a fixed dose for oral glucose tolerance tests, irrespective of body weight, for clinical determination of glucose tolerance status (World Health Organization 1980).

The question of appropriate glucose dosing applies equally to studies in animal models of obesity and type 2 diabetes. Glucose dosing based on the weight of the individual animals can result in large differences in the dose given to normal weight and obese animals. This could be a confounding factor when interpreting the result of glucose tolerance tests, in particular, in intravenous glucose tolerance tests. Proposed solutions to this issue include giving all individuals a predetermined glucose dose irrespective of weight, as is done for oral glucose tolerance testing in humans. Recently, the glucose excursions and insulin secretory responses to different regimes of oral and intraperitoneal glucose tolerance testing in mice fed normal or high‐fat diets was studied (Andrikopoulos et al. 2008). Irrespective of route of administration relatively low doses of glucose were not able to discriminate differences in glucose tolerance between normal weight and obese mice. Furthermore, fixed‐dose oral glucose tolerance testing irrespective of weight, as is done in humans, revealed significant differences between groups with regard to glucose tolerance (Andrikopoulos et al. 2008). Another recent study suggested that glucose dosing in an IVGTT in obese, leptin deficient (Ob/Ob) mice according to lean body mass is insufficient for elucidating differences in *β*‐cell function (Alonso et al. 2012).

In order to determine which glucose dosing strategy properly reveals glucose intolerance and insulin insufficiency by IVGTT, we examined mice that had been fed a chow or high‐fat diet (HFD) for 8 weeks. These mice exhibit normal and reduced insulin sensitivity, as previously reported (Winzell and Ahren 2004). In the present study, these mice were subjected to IVGTT according to different criteria: total body weight, lean body mass, or a fixed‐dose irrespective of body weight. Insulin secretion, glucose tolerance and *β*‐cell function was determined using the well‐established and widely used mathematical modeling of the IVGTT data.

## Materials and Methods

### Animals

Six‐week‐old female C57BL6/JBomTac mice were obtained from Taconic (Skensved, Denmark) and kept in a temperature controlled room (22°C) on a 12‐h light–dark cycle, with food and water ad libitum. On arrival, all mice were placed on a normal rodent diet (ND) D12450B for 1 week to acclimate to hard feed and thereafter divided into two groups and fed with ND or high‐fat diet (HFD) D12492 with 60% of kcal from fat (Lard) for 8 weeks before glucose tolerance testing. All diets were from Research Diets (New Brunswick, NJ). This study was approved by the regional animal ethical committee, Lund, Sweden.

### Body composition

Lean body mass was determined on a cohort of mice that had been fed a ND or HFD for 1, 3, 8, 12, and 16 weeks by dual emission x‐ray absorptiometry using a PIXImus imager (GE Lunar, Madison, WI). The relationship between lean body mass and total body weight was analyzed with separate linear regressions for ND or HFD fed mice. The slope and y‐intercept from the linear regressions were used to estimate the lean body mass from a given total body weight and this estimate was used for glucose dosing according to lean body mass.

### Intravenous glucose tolerance tests

IVGTTs were performed on 5 h fasted mice after 8 weeks of diet treatment. Blood samples were collected from anaesthetized mice (Hypnorm, 0.5 mg fluanisone, 0.02 mg fentanyl per mouse; Janssen, Beerse, Belgium; 0.25 mg midazolam per mouse; Dormicum, Hoffman LaRoche, Basel, Switzerland) from the retrobulbar, intraorbital, capillary plexus before d‐glucose injection (0.35 g/kg bw) to the tail vein. Additional blood samples were collected at 1, 5, 10, 20, 30, and 50 min from each mouse. Plasma was separated and stored at −20°C until it was analyzed for insulin with a mouse insulin ELISA (Mercodia, Uppsala, Sweden) and glucose (glucose oxidase method).

**Table 1.** Incremental area under the curve for intravenous glucose tolerance tests

	Total	Lean	Fixed
Glucose (mmol/L·min)			
ND	89 ± 19	59 ± 12	71 ± 14
HFD	187 ± 36^*^	105 ± 17^*^	155 ± 9^***^
Insulin (pmol/L·min)			
ND	2391 ± 984	1929 ± 1442	3861 ± 1587
HFD	4926 ± 2504	7581 ± 2337^*^	7011 ± 4470

ND, normal diet; HFD, high‐fat diet. *P <**0.05, ***P <**0.001.

### Calculations and statistics

Data are mean ± SEM unless otherwise indicated. The area under the concentration curves (AUC) was calculated with the trapezoidal rule. The glucose elimination constant (K_G_) was computed as the slope of the logarithmically transformed circulating glucose concentration between 5 and 20 min after administration of the glucose bolus. The insulin sensitivity index (S_I_) was estimated, as previously described (Pacini et al. 2001), from the analysis through the minimal model of the seven time‐point IVGTT (Pacini et al. 2001). The acute insulin response (AIR) was calculated as the mean of the suprabasal 1 and 5 min values, whereas ΔAIR was calculated as suprabasal 1 min value only. *β*‐cell function was assessed as the ratio of the suprabasal peak insulin to suprabasal peak glucose at the 1 min time point. The disposition index was calculated as S_I_ × ∆AIR. Comparisons between the different groups were performed by the Mann–Whitney *U* test. Differences were considered statistically significant when *P *<**0.05.

## Results

**Figure 1.**
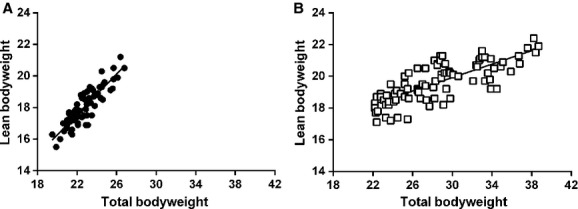
Relationship between total body weight and lean body weight. DEXA scans were used to determine the lean body weight of mice fed a (A) normal diet or (B) a high‐fat diet for 1, 3, 8, 12, or 16 weeks. Lean body weight was expressed as a function of total body weight. *N *=**77–84 per group.

**Figure 2.**
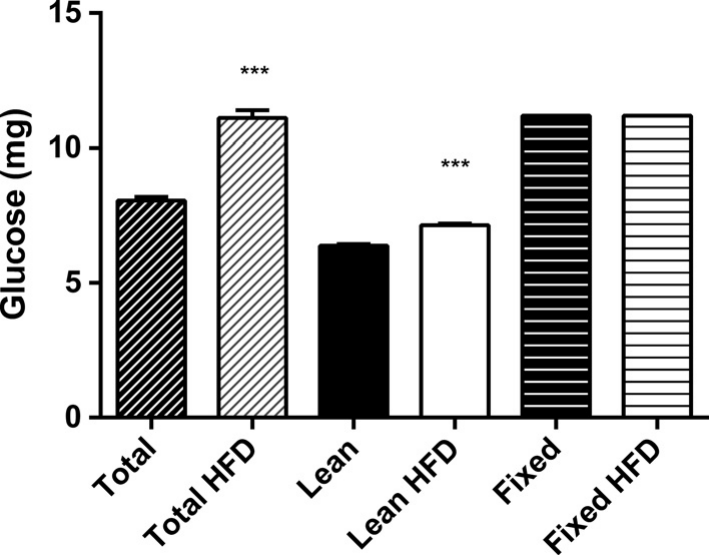
Total glucose load given during the IVGTT in each of the dosing regimens. d‐glucose was injected into the tail vein at a dose of 0.35 g/kg total body weight, lean body weight, or a fixed body weight based on the average of all experimental mice regardless of diet. *N *=**13–14 per group. ***P <**0.001.

**Figure 3.**
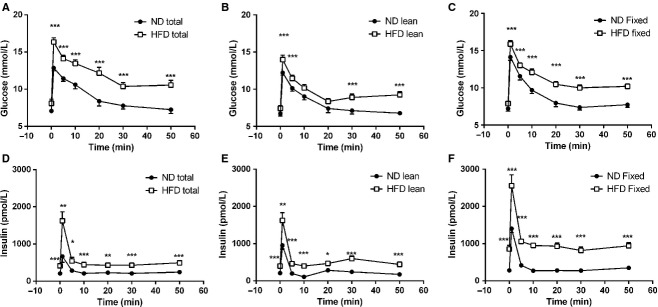
Intravenous glucose tolerance tests. Baseline blood samples were taken and 0.35 g/kg d‐glucose was injected into the tail vein. Blood samples were taken at 1, 5, 10, 20, 30, and 50 min thereafter and plasma was analyzed for glucose (A–C) and insulin (D–F). *n *=**13–14 per group. *P <**0.05, **P <**0.01, ***P <**0.001.

As expected mice fed a HFD gained more total body weight than did those fed a normal diet (data not shown). There was a positive linear relationship between total body weight and lean body mass (Fig. 1A and B). The slope of the linear regression was steeper, however, for the mice fed a normal diet (Fig. 1A). Using the linear regression data we were able to estimate the lean body mass for a given total body weight and use this information to determine the glucose dose to be given during the IVGTT. The glucose doses given based on actual total body weight, estimated lean body mass and a fixed dose based on the mean total body weight of all animals irrespective of diet are shown in Figure 2. Mice fed a HFD showed larger glucose excursions despite greater total insulin secretion than mice fed a normal diet in response to injection of glucose at a dose of 0.35 g/kg total body weight (Fig. 3A and D, Table 1). In contrast, when glucose is injected at the same dose only using lean body mass as the weight normalizer, the early glucose excursion was not significantly different to that of the normal diet mice (Fig. 3B). This was despite having elevated insulin secretion equal to that of HFD fed mice dosed based on total body weight (Fig. 3D and E).

**Figure 4.**
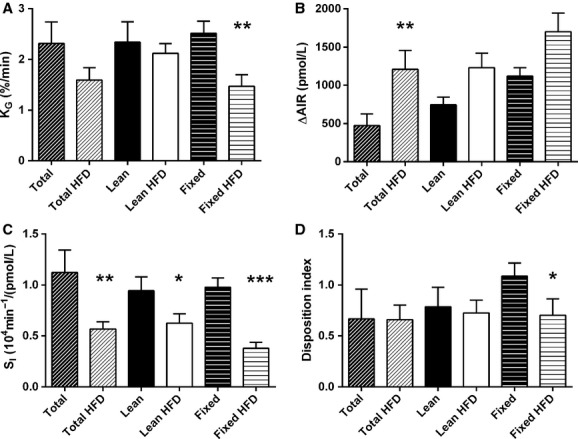
Minimal model parameters derived from IVGTT data. Glucose and insulin concentrations during the IVGTT were used to calculate the (A) glucose elimination constant K_G_, (B) acute insulin response (∆AIR), (C) insulin sensitivity index S_I_, and (D) disposition index. *N *=**13–14 per group. *P <**0.05, **P <**0.01, ***P <**0.001.

Mice were also injected with a fixed glucose dose irrespective of the weight of the individual animal. This dose was 0.35 g/mean kg body weight based on an average body weight of all animals irrespective of diet (11 mg/mouse). The fixed‐dose glucose injection resulted in hyperglycemia in the HFD fed mice with commensurate hyperinsulinemia (Fig. 3C and F) similar to HFD fed mice dosed based on total body weight. Using the glucose elimination rate constant K_G_ derived from the IVGTT data, only the fixed glucose dose strategy detected the impairment of glucose elimination induced by HFD feeding (Fig. 4A). Although all of the dosing strategies showed trends toward higher acute insulin responses (∆AIR) in the HFD mice, only the total body weight–based glucose dosing regimen resulted in statistically significant increases in ∆AIR (Fig. 4B).

**Figure 5.**
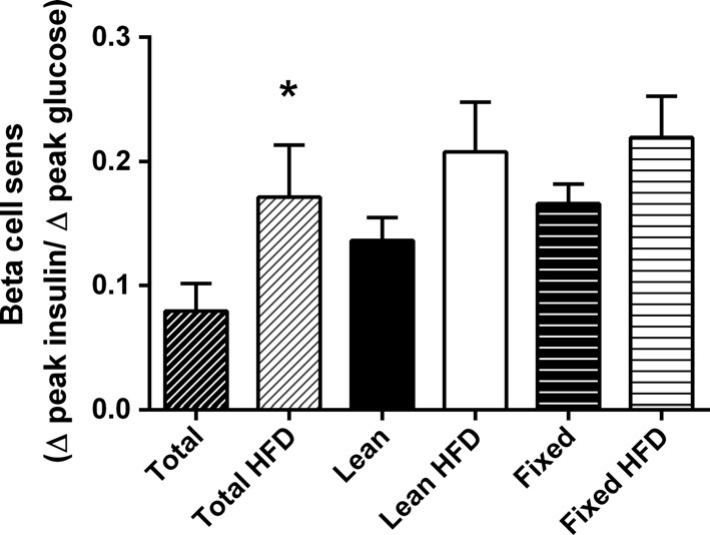
Evaluation of *β*‐cell function. The insulinogenic index was calculated from the IVGTT data as ∆ peak insulin divided by ∆ peak glucose. *N *=**13–14 per group. *P < 0.05 compared to total normal diet control.

Using the minimal model analysis of IVGTT data, we determined insulin sensitivity (S_I_) in the mice with each of the glucose dosing regimens. Each of the glucose dosing regimens demonstrated significant decreases in insulin sensitivity in mice fed a HFD (Fig. 4C). As regards the disposition index (the product of S_I_ and AIR), only the fixed glucose dose regimen was able to identify the expected impairment resulting from HFD feeding (Fig. 4D). *β*‐cell function was significantly improved in HFD fed mice when dosed according to total body weight, but not according to dosing by lean body mass or fixed glucose dose (Fig. 5).

## Discussion

The natural history of type 2 diabetes is characterized by increasing insulin resistance which is normally compensated for by increasing insulin secretion to maintain normal glucose tolerance (Bonadonna et al. 1990). If insulin resistance is inadequately compensated for due to insufficient increases in insulin secretion, hyperglycemia will occur (Ferrannini et al. 2011). The relationship between insulin sensitivity and *β*‐cell function obtained from IVGTT is described in humans by the disposition index (Kahn et al. 1993). The same concept has then been considered valid also in mice (Pacini et al. 2001). The use of the IVGTT with minimal model analysis has led to a greater understanding of the role of *β*‐cell function in the progression from normal glucose tolerance to impaired glucose tolerance and type 2 diabetes (Bergman et al. 2002). In the study presented here, the glucose dosing regimen used in the IVGTT was shown to affect the outcome and interpretation of the disposition of glucose in the HFD mice. The index showed that insulin sensitivity was impaired in HFD fed mice in all three glucose dosing regimens in agreement with sensitivity measurements derived from the euglycemic–hyperinsulinemic clamp (Pacini et al. 2001; Omar et al. 2012). The *β*‐cell sensitivity to glucose was significantly greater in HFD fed mice dosed according to total body weight, but not in HFD fed mice dosed according to lean body weight or a fixed glucose dose irrespective of body weight. There was only a trend toward increased acute insulin response in the latter groups. This is important to note as when the measures are incorporated into the disposition index, it is apparent that the increase in acute insulin response may be insufficient for the corresponding level of insulin sensitivity, resulting in a significant underestimation of the info provided by the disposition index. Only the fixed‐dose glucose dosing regimen was able to make this distinction. The other glucose dosing regimens showed no differences in the disposition index between HFD fed mice and control mice. These results are similar to the results previously demonstrated for oral glucose tolerance testing in mice where different glucose doses were tested and compared to a fixed dose (Andrikopoulos et al. 2008). In that study, a fixed glucose dose was able to effectively discriminate glucose intolerance in HFD animals, while per body weight dosing was only able to discriminate when high enough glucose amounts were used (Andrikopoulos et al. 2008).

The results expose important differences between the glucose dosing regimens which need to be considered for how the HFD model is to be related to glucose tolerance in humans. The frequently sampled IVGTT in humans is given at a dose of 0.30 g/kg total body weight, nearly identical to the 0.35 g/kg used in the mouse study presented here (Bergman et al. 1981). Dosing normal and HFD fed mice strictly in this manner revealed that the HFD fed mice had significantly reduced insulin sensitivity which was adequately compensated for by a significant increase in the acute insulin response. This resulted in a normal disposition index compared to normal chow fed animals. In contrast, when mice are given a fixed glucose dose of 0.35 g/kg, based on the mean body weight of both HFD fed and control mice, insulin sensitivity was still significantly impaired and this was not adequately compensated for by the acute insulin response which was not significantly increased, possibly due to the lower glucose dose. The conclusion was therefore a significantly impaired glucose tolerance, as determined by the glucose elimination rate, together with a significantly lower disposition index than control mice. On the other hand, dosing according to lean body mass did not reveal any differences other than a decrease in insulin sensitivity in the HFD fed mice, in agreement with results obtained in Ob/Ob mice (Alonso et al. 2012). When relating the results to the human condition one can say that risk for the development of type 2 diabetes increases as the disposition index score decreases (Bergman et al. 1981; Kahn et al. 1993). If the HFD‐induced obesity model is to be considered a model for increased risk of type 2 diabetes or “prediabetes,” then the glucose dosing strategy for IVGTT which reveals a decrease in disposition index is the most accurate to use. According to our results this would be the weight irrespective, fixed‐dose glucose dosing regimen.

## Conclusion

We thus conclude that glucose dosing regimens influence the results of insulin sensitivity and *β*‐cell function during an IVGTT, which in turn imply that careful selection of dosing regimens is important when performing studies on glucose metabolism and islet function in animals and humans for estimating diabetes risk.

## Acknowledgments

The authors acknowledge the excellent technical assistance of Kristina Andersson and the helpful discussion of the manuscript from Ulrika Axling.

## Conflict of Interest

None declared.
